# Three‐Dimensional Array Multicasting of Cylindrical Vector Beams Using Spin‐Multiplexed Dammann Vortex Metasurface

**DOI:** 10.1002/nap2.70215

**Published:** 2026-07-15

**Authors:** Zhiqiang Xie, Qingji Zeng, Huihua Huang, Zhibin Wu, Kai Niu, Junmin Liu, Shuqing Chen, Dianyuan Fan, Dingyuan Tang

**Affiliations:** ^1^ Future Technology School, College of New Materials and New Energies Shenzhen Technology University Shenzhen China; ^2^ Institute of Microscale Optoelectronics Shenzhen University Shenzhen China

**Keywords:** 3D array engineering, cylindrical vector beams, optical multicasting, spin‐multiplexed metasurface

## Abstract

Data multicasting over massive cylindrical vector beam (CVB) channels offers substantial advantages for high‐capacity information dissemination in mode‐multiplexed communication networks. However, conventional multicasting solutions are confined to one‐ or two‐dimensional operations with limited diversity; multichannel, multimode implementations in three‐dimensional (3D) vector space remain largely unexplored, which promises to maximize throughput. Herein, for the first time we develop a volumetric CVB multicasting strategy that introduces 3D multichannel array modes as information carriers. By coherently merging a vectorial Dammann vortex grating and a vectorial Dammann vortex zone plate on a minimalist spin‐multiplexed phase‐modulation metasurface, array degrees of freedom in both the transverse plane and longitudinal depth are independently regulated, thereby unlocking a 3D multichannel CVB lattice with sufficient mode diversity. As demonstration, we achieve a 3D CVB array generator with an operational dimension of 3 × 3 × 5, and 400 Gbit/s quadrature phase shift keying signals are successfully distributed to up to nine distinct spatial nodes with bit error rates of 10^−6^. This work provides a compact and scalable platform for volumetric vector‐field engineering, opening new pathways for multidimensional optical field manipulation and complex optical node interconnection, which may accelerate advances in optical edge data processing and short‐reach communication networking.

## Graphical abstract

This study demonstrates, for the first time, a 3D volumetric cylindrical vector beam (CVB) multicasting architecture that employs multichannel array modes with an operational dimension of 3 × 3 × 5 as information carriers. This approach offers substantial potential for high‐capacity information dissemination in mode‐multiplexed communication networks.

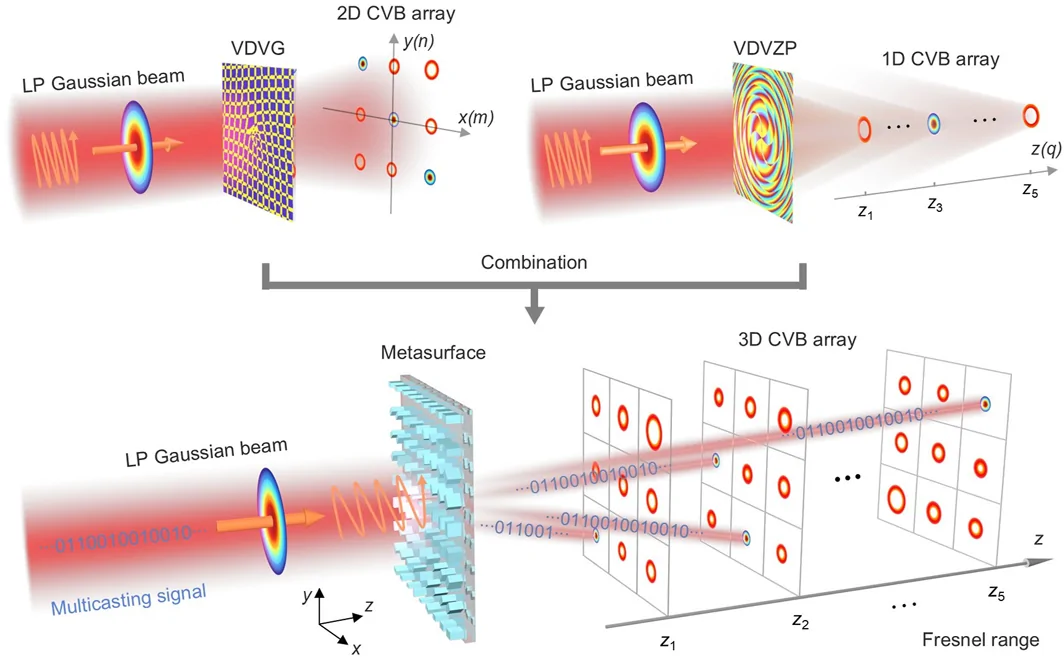

## 1 Introduction

In the deployment of optical communications and interconnections, data multicasting has evolved into an indispensable procedure for efficient information dissemination from a single source to multiple recipients, delivering substantial benefits in bandwidth conservation and latency reduction [1, 2, 3, 4]. This paradigm typically employs advanced optical channel processing technologies, such as light splitting [5, 6] and wavelength‐selective switching [7, 8], to optimize resource allocation across source‐to‐multiple‐destination paths, which thus enhances the reliability and flexibility of multinetwork interconnections. However, conventional multicasting arrangements face limitations in scalability and spectral efficiency, rooted in the underexploited photon dimensionality and impending bandwidth exhaustion in wavelength division multiplexing. The recent advent of cylindrical vector beams (CVBs) is instigating a paradigm shift in optical communications and interconnections by harnessing their orthogonally independent vector modes across inhomogeneous polarization structures [9, 10, 11, 12, 13]. This revolutionary approach develops the photon's spatial degrees of freedom to transcend current bandwidth constraints through mode division multiplexing, opening new windows for information carriage, transmission, and processing [14, 15, 16, 17, 18, 19]. Although extensive research on CVB manipulation has propelled advances in multiplexing/demultiplexing [20], cross‐connection [21], and add‐drop multiplexing [22] functionalities, a multichannel multimode multicasting operation remains largely unresolved. It is dedicated to achieving multinode information transfer within a volumetric space for increased spatial bandwidth capacity purposes, holding great potential in edge data centers and short‐reach networking communications.

Engineering the multicasting architectures using arrays of multiple CVBs boosts multimode parallel processing capabilities and amplifies data channel density, promising to unlock high‐capacity, multispot, and multiview application scenarios [23, 24, 25]. Most prevalent and straightforward schemes for creating such multibeam arrays involve continuously stacking spiral and gradient/lens phase profiles onto a single optical element [26, 27, 28], permitting independent tuning of individual beam modes and spatial positioning. Although this methodology facilitates one‐ and two‐dimensional (1D/2D) spatial projects of scalar vortices or CVBs [29, 30, 31, 32], excessive phase superposition inadvertently induces pronounced diffraction scattering in an absence of amplitude modulation, thereby resulting in low mode‐field conversion efficiency and uneven energy distribution across beam channels. Recent progress in periodic phase structures mitigates this issue via grating‐based encoding strategies, which employ binary phase modulation to ensure uniform energy layout in 1D/2D vector fields while suppressing scattering losses [33, 34, 35, 36, 37, 38, 39]. Nevertheless, this method generally strives for diffraction‐order‐independent topological modulation, leading to limited mode diversity over multichannel CVBs. Furthermore, current implementations remain constrained to planar configurations, restricting multichannel CVB operations to 1D/2D spatial domains. Achieving multichannel multimode arrays in three‐dimensional (3D) space—essential for maximizing multicasting channel capacity and supporting multiuser interconnection environments—is still a persistent challenge.

In this research, we present an approach for the volumetric generation of multichannel, multimode CVBs using a spin‐multiplexed Dammann vortex metasurface, and affirm its utility in 3D array multicasting. Profiting from spin‐multiplexed phase modulation [40, 41], a vectorial Dammann vortex grating (VDVG) is encoded into the metasurface to construct a 2D in‐plane CVB array featuring centrosymmetric mode conjugation, and a vectorial Dammann vortex zone plate (VDVZP) is concurrently incorporated to yield a 1D longitudinal CVB array with axial symmetric mode conjugation. The synergistic combination of these two elements enables multichannel, multimode operations in a 3D vector space within the Fresnel region, producing a volumetric array with spatially varying polarization topology formats across its diffraction orders. As a proof‐of‐principle demonstration, a 3D CVB engineering was realized with an array operational dimension of 3 × 3 × 5. In a dedicated optical multicasting communication system, 400 Gbit/s quadrature phase shift keying (QPSK) signals were multicast to eight or nine distinct 3D spatial ports via a selective switching of incident polarization order, attaining the bit error rates (BERs) approaching 1 × 10^−6^. By optimizing the Dammann vortex element parameters in both transverse and longitudinal dimensions, a greater number of multicasting channels can be accommodated, thereby creating an expanded volumetric vector diffraction space. Overall, the proposed CVB array generation scheme affirms the advantages of multichannel, multimode, and volumetric engineering, which may unlock more possibilities for sophisticated manipulation of multidimensional vector optical fields and their advanced applications beyond multicasting communications.

## 2 Principle and Design

The 3D CVB array we propose for multicasting signals is constructed through the multiplicative synthesis of a 2D planar array and a 1D axial array, giving rise to diverse multivectorial textures across a multichannel diffraction space. To enhance mode diversity in the *x*–*y* transverse plane while ensuring uniform energy distribution across diffraction channels, a VDVG is designed to generate CVBs arranged in a 2D grid, as depicted in Figure 1a. Specifically, this grating element utilizes spin‐multiplexed phase modulation to couple two independent binary scalar DVG phase profiles (see Supporting Information S1: Note S1), which are spatially segregated to manipulate the left‐ and right‐handed circularly polarized (LHCP/RHCP) components of incident light, thereby enabling off‐axis vector control for in‐plane multichannel CVBs. The transmission function of the VDVG corresponding to the LHCP and RHCP components in Cartesian coordinates (*x*, *y*) can be mathematically formulated as follows:

(1) $$T_{\text{VDVG}}^{L/R}\left(x,y\right)=\sum_{m=-M/2}^{M/2}\sum_{n=-N/2}^{N/2}{\left|C_{mn}\right|}^{2}\exp\left[im\left(\frac{2\pi x}{\Lambda_{x}}\pm \Delta l_{x}\theta \right)+in\left(\frac{2\pi y}{\Lambda_{y}}\pm \Delta l_{y}\theta \right)\right]\left[\begin{array}{l} 1\\ \mp i \end{array}\right],$$

where (*m*, *n*) denotes the diffraction order in the *x*–*y* plane, with *m* ranging from −*M*/2 to *M*/2 and *n* from −*N*/2 to *N*/2. *M* and *N* here represent the total number of diffraction orders along the *x* and *y* directions, respectively. The coefficient |*C*_*mn*_|^2^ corresponds to the normalized energy of the (*m*, *n*)th diffraction order relative to the total power. The parameters Λ_*x*_ and Λ_*y*_ define the grating phase periods along the *x*‐ and *y*‐axes, whereas Δ*l*_*x*_ and Δ*l*_*y*_ denote the topological intervals in the respective directions. The azimuthal angle is given by *θ* = arctan(*y*/*x*). The LHCP and RHCP components correspond to Jones vectors: |*L*〉 = [1 −*i*]^*t*^, |*R*〉 = [1 *i*]^*t*^, and *t* is matrix transpose operator. Based on this grating structure, the resulting output optical field response under illumination by a linearly polarized (LP) Gaussian beam (comprising both LHCP and RHCP components) can be derived as follows:

(2) $$T_{\text{VDVG}}^{\text{LP}}\left(x,y\right)=\sum_{m=-M/2}^{M/2}\sum_{n=-N/2}^{N/2}{\left|C_{mn}\right|}^{2}\exp\left(im\frac{2\pi x}{\Lambda_{x}}+in\frac{2\pi y}{\Lambda_{y}}\right)\left[\begin{array}{l} \cos\left[\left(m\Delta l_{x}+n\Delta l_{y}\right)\theta +\vartheta \right]\\ \sin\left[\left(m\Delta l_{x}+n\Delta l_{y}\right)\theta +\vartheta \right] \end{array}\right],$$

in which *ϑ* represents the initial polarization orientation. According to Equation (2), an (*M* + 1) × (*N* + 1) array of CVBs is obtained across the output diffraction region, as illustrated in Figure 1a. As a demonstration of this work, we define *M* = *N* = Δ*l*_*x*_ = Δ*l*_*y*_ = 2 and *ϑ* = 0. This configuration results in a polarization order of (*m*Δ*l*_*x*_ + *n*Δ*l*_*y*_) for the CVB at the (*m*, *n*)th diffraction order, thereby producing a rectangular 2D cylindrical vector array exhibiting centrosymmetric mode conjugation in the *x–y* transverse output plane.

**FIGURE 1:**
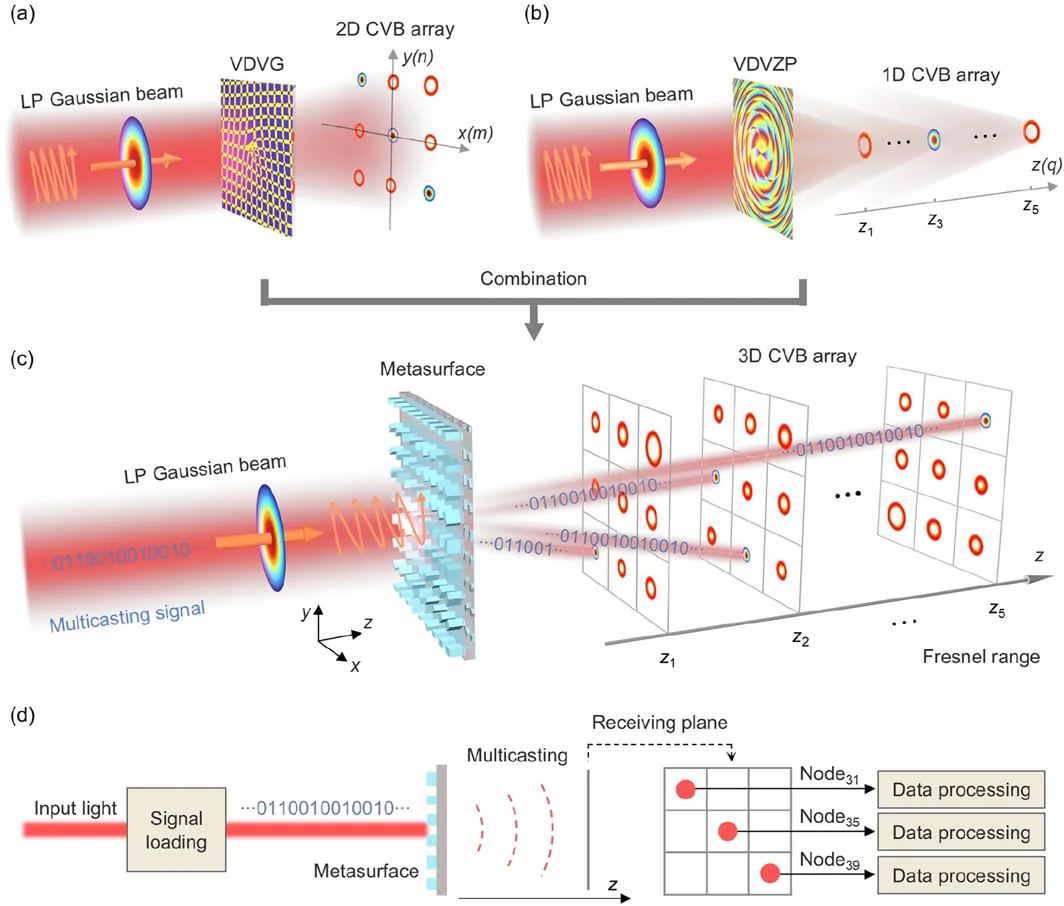
Working mechanism and design of 3D CVB array. (a) Generation of a 2D planar CVB array using a LP Gaussian beam to irradiate the VDVG element. (b) Formation of a 1D axial CVB array utilizing a LP Gaussian beam to illuminate the VDVZP device. (c) Creation of a multichannel, multimode 3D CVB array by merging the VDVG and VDVZP components into a spin‐multiplexed metasurface. (d) The operating mechanism of metasurface‐enabled multinode data multicasting processing.

To achieve diversified arrangement of CVBs along the axial propagation depth, a VDVZP is introduced, as described in Figure 1b. This element incorporates a pair of spin‐dependent binary scalar DVZP phase profiles and a shared lens phase (see Supporting Information S1: Note S1), establishing on‐axis vector control for longitudinal multifoci CVBs. The transmission function of the VDVZP corresponding to the LHCP and RHCP components in polar coordinates (*r*, *φ*) is mathematically expressed as follows:

(3) $$T_{\text{VDVZP}}^{L/R}\left(r,\varphi \right)=\sum_{q=-Q/2}^{Q/2}{\left|C_{q}\right|}^{2}\exp\left[iq\left(\frac{2\pi }{\Lambda_{z}}\sqrt{1-{\left(r\;\sin\;\alpha \right)}^{2}}\pm \Delta l_{z}\varphi \right)\right]\times \exp\left(i\frac{kr^{2}}{2f}\right)\left[\begin{array}{l} 1\\ \mp i \end{array}\right],$$

where *q* denotes the diffraction order along the *z*‐axis, ranging from −*Q*/2 to *Q*/2, and *Q* represents the total number of diffraction orders in the *z*‐direction. |*C*_*q*_|^2^ corresponds to the Fourier coefficient of the *q*th diffraction order, associated with the phase transition point within one period Λ_*z*_. The parameter *α* is related to the numerical aperture (NA) of the zone plate as *α* = arcsin(NA), and Δ*l*_*z*_ defines the topological interval along the *z*‐direction. *k* is the wavevector, and *f* is the focal length. Under illumination by a LP Gaussian beam, the resulting output optical field response can be expressed as follows:

(4) $$T_{\text{VDVZP}}^{\text{LP}}\left(r,\varphi \right)=\sum_{q=-Q/2}^{Q/2}{\left|C_{q}\right|}^{2}\exp\left[iq\left(\frac{2\pi }{\Lambda_{z}}\sqrt{1-{\left(r\;\sin\;\alpha \right)}^{2}}\right)\right]\times \exp\left(i\frac{kr^{2}}{2f}\right)\left[\begin{array}{l} \cos\left(q\Delta l_{z}\varphi +\vartheta \right)\\ \sin\left(q\Delta l_{z}\varphi +\vartheta \right) \end{array}\right].$$

As seen in Figure 1b, a 1 × (*Q* + 1) coaxial CVB array can be reconstructed within the Fresnel diffraction region, wherein the polarization order of beam at the *q*th diffraction order is *q*Δ*l*_*z*_ (Here, we stipulate *Q* = 4, Δ*l*_*z*_ = 2, and *ϑ* = 0 in this work), thus creating a 1D longitudinal cylindrical vector array with axial symmetric mode conjugation along the propagation direction.

The independence of phase modulation allows the transmission functions of the VDVG and VDVZP to be linearly combined and integrated into a monolithic compact spin‐multiplexed metasurface, with a resultant transmission function of $T_{\text{VDVG}}^{L/R}\times T_{\text{VDVZP}}^{L/R}$. This design forms a 3D CVB array with the operational dimension of (*M* + 1) × (*N* + 1) × (*Q* + 1), featuring concurrent multichannel and multimode operations, as indicated in Figure 1c. Detailed parameters and the spin‐resolved phase functions for vector field synthesis are provided in Supporting Information S1: Note S1. Consequently, this engineered 3D vector light field establishes a platform for amplifying multicasting channel capacity and dispensing multiple digital signal streams within a Fresnel volumetric space, thereby supporting multinode interconnections with mode division multiplexing. Figure 1d outlines the underlying mechanism of multinode data processing. Chronologically, the input light, perpendicularly incident on the metasurface, undergoes initial digital signal loading via a modulator, and is subsequently delivered to designated spatial nodes across distinct depth planes through 3D CVB diffractive multicasting. At the specific receiving nodes, the optical signals are intercepted and converted by photodetectors for subsequent data demodulation (see Supporting Information S1: Note S5 for details). This architecture therefore enables efficient information interconnections across diverse multidepth planes and multispatial nodes.

The spin‐multiplexed phase modulation mechanism in meta‐optics enables independent control of the two spin components of light, thereby allowing the imposition of desired phase profiles Ψ_*L*_(*x*, *y*) = arg($T_{\text{VDVG}}^{L}\times T_{\text{VDVZP}}^{L}$) and Ψ_*R*_(*x*, *y*) = arg($T_{\text{VDVG}}^{R}\times T_{\text{VDVZP}}^{R}$) on the LHCP and RHCP components to realize 3D CVB engineering. Specifically, the modulation satisfies |*L*〉 → exp[*i*·Ψ_*L*_(*x*, *y*)]|*R*〉 and |*R*〉 → exp[*i*·Ψ_*R*_(*x*, *y*)]|*L*〉 after the incident wave transmits through the metasurface. Following the synchronous modulation principle of propagation and Pancharatnam–Berry phase [42, 43], a series of meta‐atoms with varying in‐plane dimensions—functioning as subwavelength half‐wave plates with high transmission efficiency and full 2π phase coverage at the operating wavelength—are arranged at specific spatial rotation angles to construct the metasurface capable of delivering such spin‐multiplexed phase modulation. The required phase shifts and rotation angles linking phase profiles Ψ_*L*_(*x*, *y*) and Ψ_*R*_(*x*, *y*) can be derived as follows:

(5) $$\begin{array}{l} \varphi_{x}\left(x,y\right)=\left[\Psi_{L}\left(x,y\right)+\Psi_{R}\left(x,y\right)\right]/2\\ \varphi_{y}\left(x,y\right)=\left[\Psi_{L}\left(x,y\right)+\Psi_{R}\left(x,y\right)\right]/2-\pi \\ \gamma \left(x,y\right)=\left[\Psi_{L}\left(x,y\right)-\Psi_{R}\left(x,y\right)\right]/4 \end{array}.$$

As such, at each point (*x*, *y*) of the metasurface, the propagation phases (*φ*_*x*_, *φ*_*y*_) and rotation angle *γ* of the meta‐atom can be determined from the target phase profiles (Ψ_*L*_, Ψ_*R*_), thereby empowering spin‐multiplexed manipulation of two distinct phase impositions on orthogonally circular‐polarized incident light for CVB array control.

Based on the above principles, we demonstrate the spin‐multiplexed metasurface engineered through a periodic array of silicon (Si) meta‐atoms, as illustrated in Figure 2a. Each unit structure is composed of a Si meta‐atom positioned on a square silicon dioxide (SiO_2_) substrate, with its oblique and top views shown in the dashed box. For multicasting operation at a wavelength of 1550 nm, the lattice period (*P*) of the unit cells is consistently defined as 800 nm to access the subwavelength condition, which is capable of suppressing high‐order diffraction while satisfying the Nyquist sampling criterion. The height (*H*) of the Si meta‐atom is optimized to 800 nm to enable full 0‐to‐2π phase coverage. Such structure design simultaneously facilitates the optimization of array layouts via subwavelength‐scale light‐field unit tuning, while guaranteeing Mie resonance‐supported, low‐loss transmissive wavefront manipulation within a dielectric framework [44, 45]. To precisely determine the unit parameters tailored for spin‐multiplexed phase modulation, we performed full‐wave simulations using the finite‐difference time‐domain (FDTD) method on Si meta‐atoms with varying length (*L*) and width (*W*). The amplitude (*A*_*x*_, *A*_*y*_) and phase (*φ*_*x*_, *φ*_*y*_) responses as functions of *L* and *W* are summarized in Figure 2b. From this FDTD‐precomputed meta‐atom library, we selected eight Si meta‐atom unit structures, comprising four basic geometries and their mirrored counterparts, with specific *L* and *W* values of S_1_ (380 nm, 260 nm), S_2_ (400 nm, 270 nm), S_3_ (460 nm, 280 nm), S_4_ (200 nm, 410 nm), S_5_ (260 nm, 380 nm), S_6_ (270 nm, 400 nm), S_7_ (280 nm, 460 nm), S_8_ (410 nm, 200 nm). This configuration effectively discretizes the full 0–2π phase space into eight steps while maintaining a π phase shift (i.e., |*φ*_*x*_ − *φ*_*y*_|) between the *x*‐ and *y*‐polarizations, as showcased in Figure 2c. To further verify the half‐wave behavior of the selected structures, we simulated the phase delay of the four basic units under LHCP illumination as a function of the in‐plane rotation angle *γ*. As illustrated in Figure 2d, the phase delay in all cases varies linearly with twice the rotation angle, confirming that each meta‐atom satisfies the half‐wave condition necessary for Pancharatnam–Berry phase modulation. Additionally, the transmittance (T) and polarization conversion efficiency (PCE) of the four basic unit structures under LHCP illumination were numerically evaluated, with the results outlined in Figure 2e. Here, the PCE is defined as the intensity ratio of the cross‐circularly polarized output to the total transmitted field. These results confirm that the Dammann vortex metasurface composed of the eight selected Si meta‐units is poised to enable efficient light‐field manipulation with acceptable optical loss, thereby offering a robust platform for constructing the metasurface‐based multicasting devices.

**FIGURE 2:**
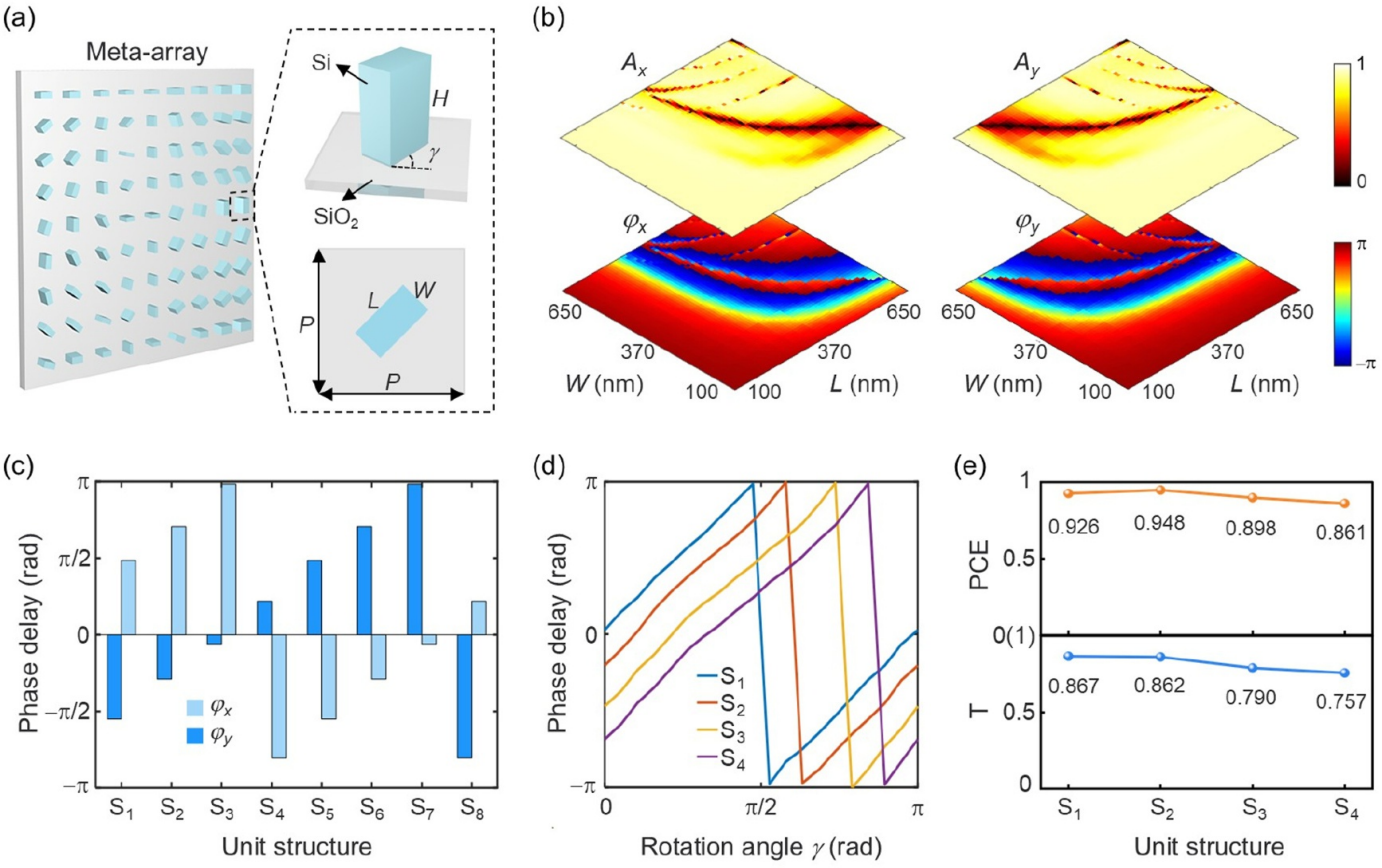
Design of the spin‐multiplexed Dammann vortex metasurface. (a) Left: Schematic of the desired metasurface array; right: oblique and top views of a Si meta‐atom unit structure placed on a square SiO_2_ substrate. (b) Numerical characterization of the amplitude (*A*_*x*_, *A*_*y*_) and phase (*φ*_*x*_, *φ*_*y*_) responses versus Si meta‐atoms’ *L* and *W*, varied in 10 nm increments from 50 to 700 nm. (c) Propagation phase delay (*φ*_*x*_, *φ*_*y*_) of the eight chosen Si meta‐atoms. (d) Phase delay curves of four basic Si units as a function of rotation angle *γ* under a 1550 nm LHCP light. (e) T and PCE coefficients of four basic Si units at the operating wavelength of 1550 nm.

## 3 Results and Discussion

In a proof‐of‐principle experimental demonstration, we designed a metasurface‐based 3 × 3 × 5 CVB array generator leveraging the spin‐multiplexed Dammann vortex phase modulation mechanism. The meta‐device was fabricated via standard electron‐beam lithography and subsequent lift‐off processes, with detailed fabrication procedures provided in Supporting Information S1: Note S2. The fabricated metasurface comprises a 1000 × 1000 array of individually addressable Si meta‐atoms arranged within an 800 × 800 μm^2^ footprint, with structural details confirmed by scanning electron microscopy (SEM) in Figure 3a. To validate the device's performance in 3D CVB engineering, we constructed a dedicated optical setup, as presented in Figure 3b. A laser source centered at 1550 nm emitting a collimated Gaussian beam was used as the input. A half‐wave plate (HWP) followed by a linear polarizer (LP) were employed to regulate the incident polarization to linear and adjust the optical intensity to prevent overexposure. Prior to interacting with the meta‐device, the beam was condensed by a lens with a focal length of 200 mm to increase energy density and confine the illumination within the active area of the sample. The resulting diffracted optical fields were magnified using a 20 × objective lens (OL) and recorded by a translating charge‐coupled device (CCD) detector at various axial planes. Moreover, an optional LP placed in front of the CCD allowed analysis of the vectorial profiles of the output CVB arrays.

**FIGURE 3:**
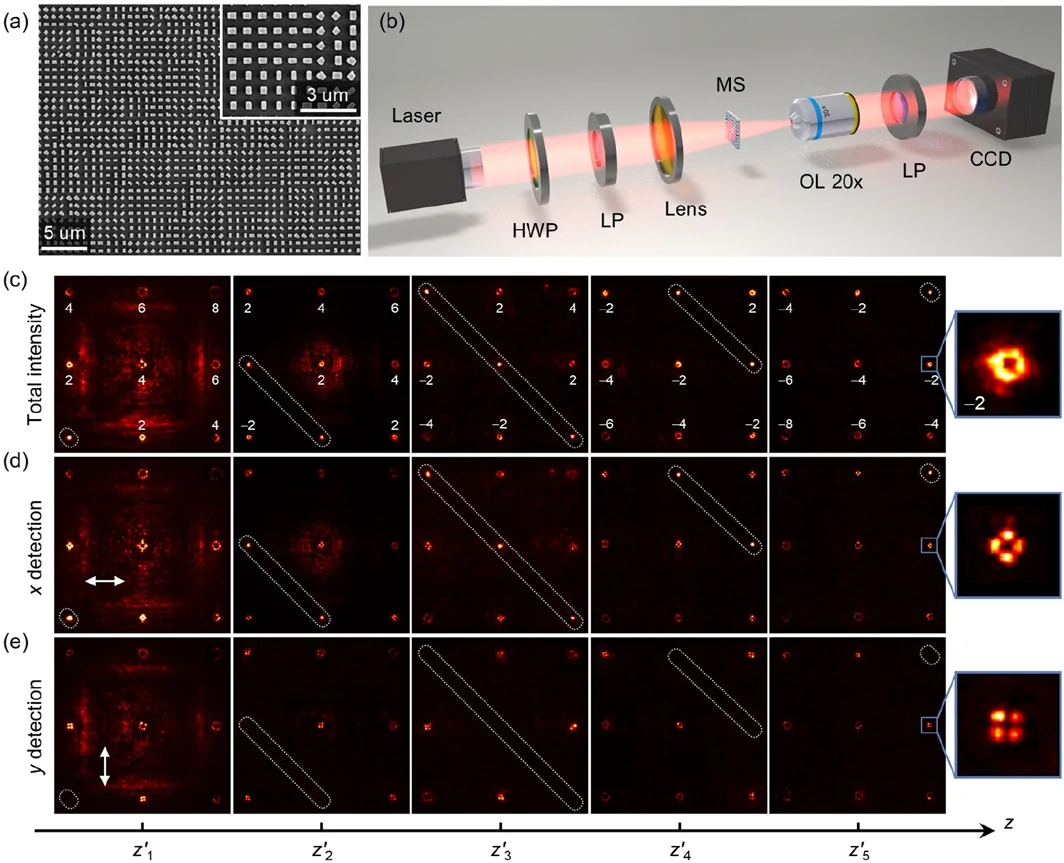
Optical characterization of the fabricated metasurface‐based 3 × 3 × 5 CVB array generator. (a) SEM images of the fabricated sample. (b) Experimental setup for evaluating the optical performance of the metasurface (MS). The experimentally captured 3D CVB array comprises 3 × 3 sub‐arrays with distinct polarization orders distributed across five *z*‐direction propagation depths, showing the corresponding (c) total intensity, (d) *x*‐polarized, and (e) *y*‐polarized detection results. The white double arrow indicates the direction of polarization.

Under Gaussian beam illumination, Figure 3c displays a set of 2D 3 × 3 cylindrical vector patterns with spatially varying polarization orders across five propagation depths ($z_{1}^{\prime }$ to $z_{5}^{\prime }$), leading to the volumetric generation of a 3 × 3 × 5 CVB array in the Fresnel diffraction space. A clear diversity in polarization order is observed across each transverse plane along the propagation direction (*z*‐axis), and a finding further corroborated by the *x*‐ and *y*‐polarized detection results shown in Figure 3d,e, respectively. The specific polarization order values for each CVB located at the corresponding node of the 3D lattice are tabulated in Figure 3c, consistently matching the indexing results documented in Supporting Information S1: Note S3. Notably, axial displacement of the CCD camera enables depth‐resolved capturing of CVB array evolution, where discrepancies between experimental observation planes and theoretical predictions arise from OL‐induced light field magnification effects. In other words, distances between the receiving planes and the metasurface sample differ from those used in theoretical calculations. Despite this disparity, the 3D CVB engineering exhibits good agreement between experimental measurements and simulation results. In the figures, white‐dashed boxes indicate positions where the CVB polarization order equals zero. These nodes can be regarded as direct receiver channels for multicast users, as the beam at these locations adopts a fundamental Gaussian mode that allows direct signal demodulation. For other regions with nonzero polarization orders, information multicasting and access necessitate back‐conversion into the fundamental Gaussian mode by illuminating the corresponding conjugate‐order CVB. Furthermore, we analyzed the operational bandwidth of the proposed CVB array generation system, demonstrating an 83 nm bandwidth window capable of supporting a robust broadband response; the underlying effects and detailed evaluation results are outlined in Supporting Information S1: Note S4. These results reveal the successful generation of multichannel, multimode CVB arrays in 3D space, establishing a physical platform for multinode, multiuser signal interconnections in a volumetric multicasting setting.

Theoretically, Dammann elements optimize the energy distribution across diffraction orders to achieve uniform intensity. To reveal this performance, we utilized the experimentally obtained 2D CVB array patterns (arranged at five distinct depths) as inputs, and designed a series of single‐aperture masks to selectively isolate the CVB subchannels at individual lattice sites. The energy per diffraction order was then determined through offline calculation of the effective optical field intensity. This processing workflow can be found in Figure 4a. Figure 4b presents the normalized power values of each valid CVB channel within each transverse plane, comparing simulation results with experimental measurements. Notably, the energy fluctuation among diffraction orders is less than 19.57% in these simulated optical fields, whereas this fluctuation reaches a maximum of 76.78% in experimental measurements. Potential contributors to this experimental deviation may involve spectral overlap at the coaxial planes and scattering interference among arrays in both transverse and longitudinal dimensions, which inevitably arise from the finite apertures and optical elements (e.g., the objective lens and iris) used in the experimental setup. Additional contributing factors include imperfect phase modulation and polarization conversion of the fabricated metadevice, attributable to nanofabrication imperfections such as sidewall roughness and critical dimension errors, as well as potential slight experimental misalignments between the incident beam, metasurface, and detector. Despite these nonidealities in channel power uniformity, the generated multichannel. multimode CVB fields still exhibit strong agreement with the theoretical design, thereby laying a solid foundation for multinode multicasting communication with mode division multiplexing.

**FIGURE 4:**
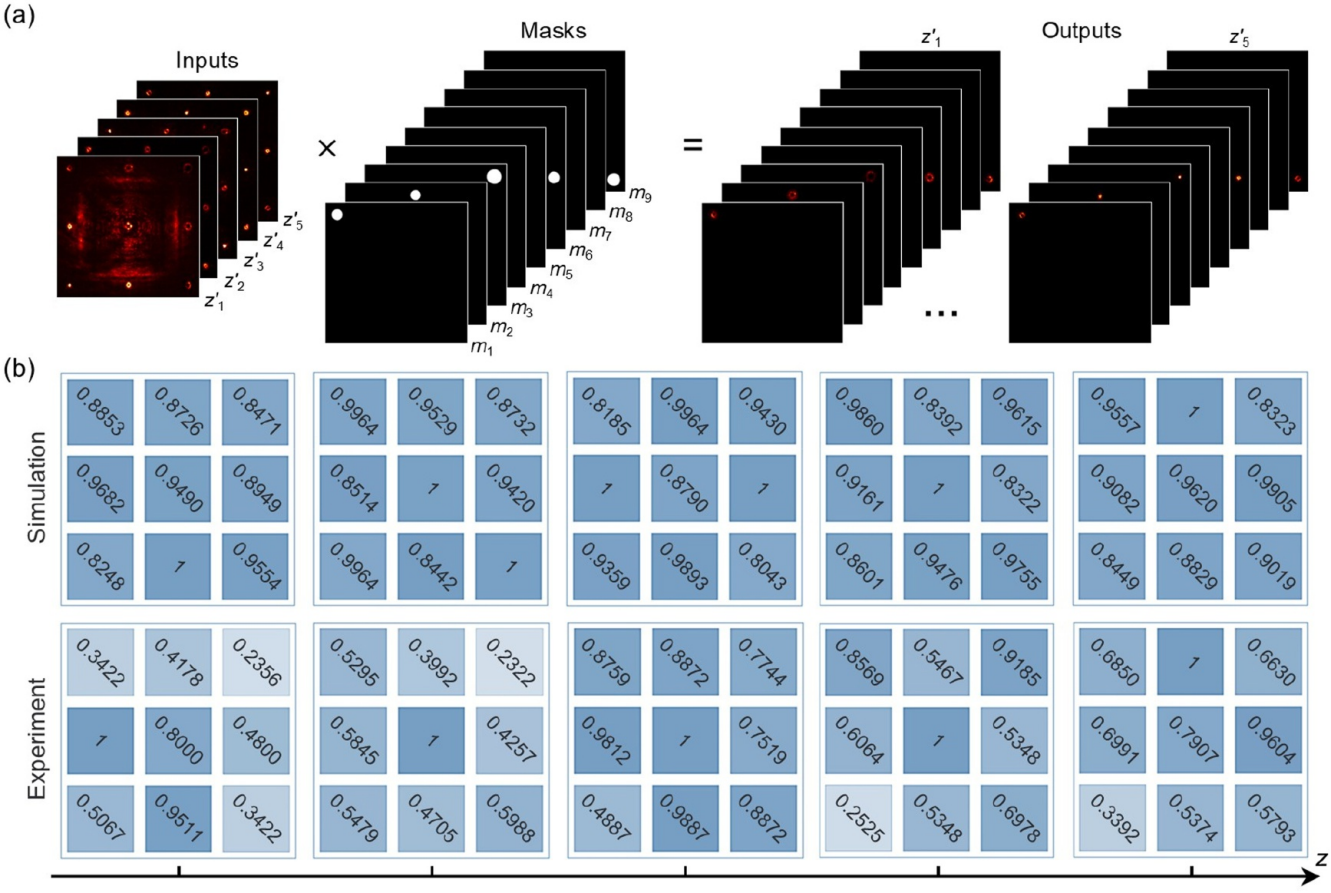
Quantitative characterization of the effective energy per CVB channel across transverse planes at different depths. (a) Workflow for calculating the energy of the experimentally generated 3D vortex array at each node of the 3D lattice. (b) Normalized power of each effective CVB channel within each depth, showing a comparison between simulations and experiments.

To demonstrate the practical efficacy of the proposed 3 × 3 × 5 CVB array generator for 3D multinode multicasting interconnections, we developed a dedicated optical multicasting communication prototype, which is detailed in Supporting Information S1: Note S5. Incorporated with wavelength division multiplexing and polarization division multiplexing technologies, 400 Gbit/s QPSK signals were modulated onto preconfigured input CVBs (with polarization orders of 0 or +2), serving as a single‐channel data source. The designed metasurface‐based 3D CVB array device enabled spatial volumetric replication of the seed optical signals, realizing one‐to‐multinode information multicasting within the Fresnel diffraction region. This configuration would reflect the metadevice's utility for short‐reach data interconnections and access. Figure 5a,b present the performance of free‐space 3D multicasting communication when the input sources are CVBs with polarization orders of 0 and +2, respectively, characterized by BER curves and constellation diagrams. Notably, we designate the positions of diffraction orders retrieved back to Gaussian beams within the diffracted 3D CVB lattice as multicasting signal access ports. Only those diffraction orders satisfying the conjugate‐mode matching condition are converted back to fundamental Gaussian beams. Signals carried by these fundamental Gaussian beams can therefore be directly coupled into optical fibers for demodulation without requiring additional mode conversion. This seamless integration is perfectly compatible with standard coherent optical detection schemes, thereby providing multicasting users with a direct, high‐fidelity access channel to retrieve signal content.

**FIGURE 5:**
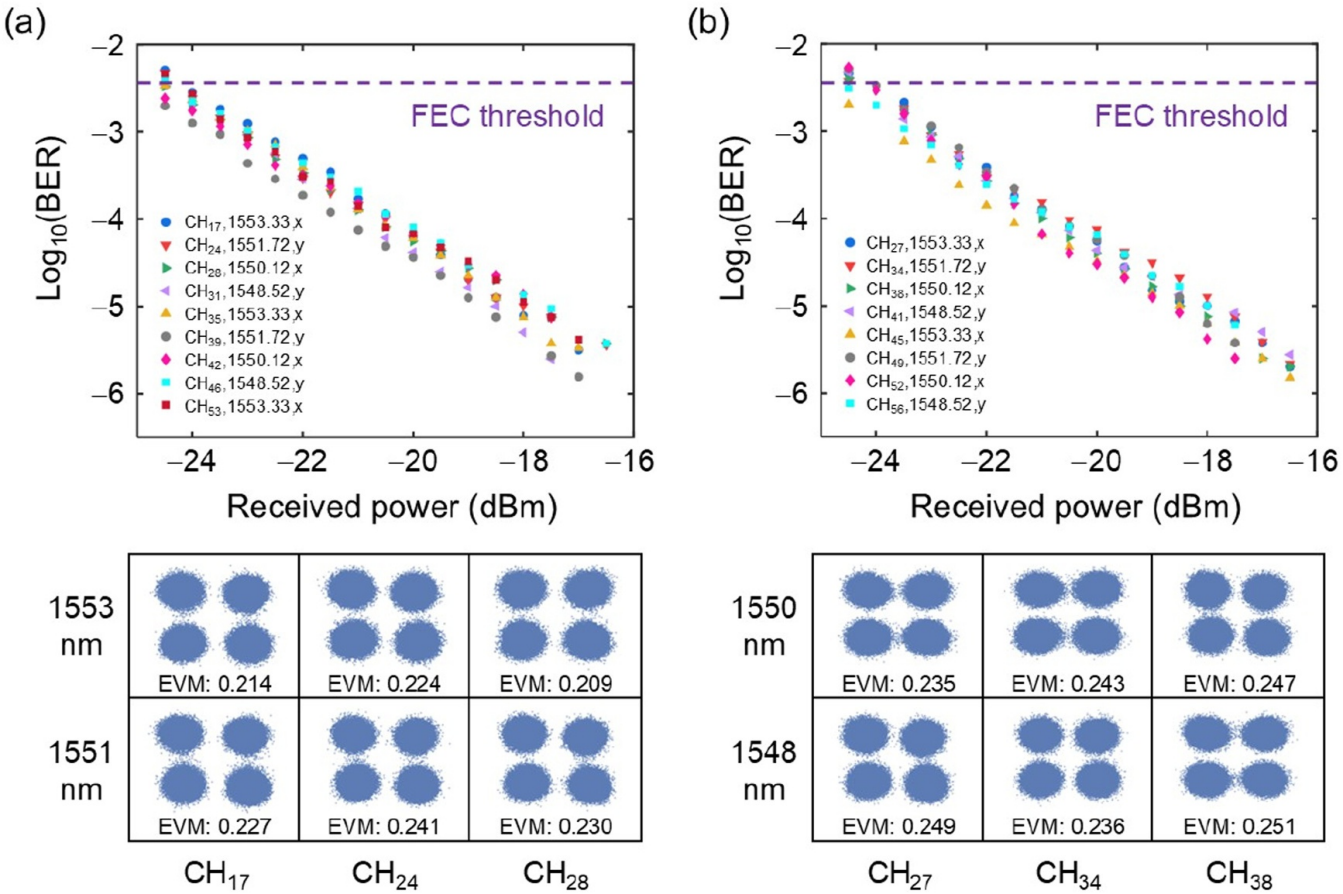
Communication outcomes of data dissemination using 3D CVB array multicasting. Measured BER curves and constellation diagrams upon signal multicasting using the (a) 0‐polarization‐order CVB and (b) +2‐polarization‐order CVB input source.

For these access ports under the 0‐polarization‐order CVB incidence, Figure 5a extracts and presents several BER curves of the nine channels as a function of received optical power. Here, a label such as “CH_*ab*_, 1553.33, *x*” denotes the channel corresponding to the signal received from the *b*th port at the *a*th diffraction depth plane, operating at a wavelength of 1553.33 nm and *x*‐polarization dimension. The index *b* is assigned according to a top‐to‐bottom, left‐to‐right priority within the transverse plane; other labels follow the same convention. The results show that all BERs fall below the hard‐decision forward error correction (FEC) threshold of 3.8 × 10^−3^ when the received optical power exceeds −24 dBm. The slight channel‐to‐channel BER variations mainly arise from the nonuniform energy distribution among diffraction orders, residual mode impurities from imperfect polarization conversion, wavelength‐dependent power imbalances across the wavelength‐division multiplexed channels and erbium‐doped fiber amplifier gain profiles, as well as minor experimental misalignments and interorder scattering interference. Despite this, as the received power increases, the BERs for all channels gradually approach 1 × 10^−6^. The sampled constellation diagrams of selected channels demonstrate similar and convergent characteristics at a received optical power of −19 dBm, their corresponding error vector magnitudes (EVMs) are less than 0.23. To visualize the one‐to‐many data replication capability in this multicasting implementation, Supporting Information S1: Note S6 presents the measured temporal waveforms of the input source and the postmulticasting receiver channels, demonstrating successful signal dissemination. Comparable multicasting effectiveness is also validated under illumination with a +2‐polarization‐order CVB, where signals are successfully distributed to eight nodes within the volumetric space, as shown in Figure 5b. This nearly linear log‐scale decrease of BER with increasing received optical power is consistent with the exponential decay of bit error probability as a function of signal‐to‐noise ratio in an additive white Gaussian noise (AWGN) channel, indicating that the multicasting link operates under Gaussian‐noise‐limited conditions without significant nonlinear distortions. Although crosstalk among channels exists due to spectral overlap and imperfect metasurface fabrication, its level remains sufficiently low that it does not dominate the noise budget, as evidenced by the well‐converged constellation diagrams and the AWGN‐like linear BER trend with received optical power. Furthermore, Supporting Information S1: Note S7 provides an extended evaluation of the multicasting communication performance under ± 4‐polarization‐order CVB inputs; the data confirm that, although the spatial configuration of the access ports shifts according to the conjugate‐mode matching condition, the overall BER performance remains highly comparable across all tested orders. These results not only confirm the robust excellent data‐dissemination performance of the communication system but also underscore the strong potential of the proposed 3D array multicasting scheme for deployment in short‐reach data interconnections.

CVB arrays provide an enhanced space‐bandwidth product, maximizing the number of data channels and capacity in optical multicasting communications through multichannel engineering of diverse vector modes. By introducing a spin‐multiplexed Dammann vortex metasurface, our work successfully realizes spatial deployment of multichannel, multimode vector beams in a full 3D layout. This achievement enables a critical dimensionality transcendence from existing scalar vortices [46] to complex vector fields, unlocking a volumetric vector architecture that, for the first time, empowers multinode data multicasting within a 3D vectorial domain. Our demonstration focuses on a 3D CVB array generator with an operational dimension of 3 × 3 × 5; however, the channel scale can be further expanded—both in the transverse and longitudinal planes—by optimizing Dammann structural parameters to access a larger volumetric vector diffraction space (see Supporting Information S1: Note S8). Notably, configuring large‐scale multicasting nodes will cause substantial division of the incident optical power, thereby limiting the signal strength allocated to each receiving channel. This may introduce instability to signal propagation in free space and accurate demodulation in optical fibers. Thus, a practical trade‐off exists between the number of multicasting channels and the received signal intensity. On the other hand, although our work is demonstrated in the near‐infrared 1550 nm regime, the proposed array multicasting design can be extended to visible [47] or midinfrared [48] windows by substituting the metasurface materials, which may offer a versatile platform for visible/midinfrared multicasting interconnection scenarios. We anticipate that this work will benefit optical edge data processing tasks such as synchronized distribution of neural network weights from a central server to multiple edge nodes (e.g., robotic arms or smart cameras) in a data center or factory floor, as the ability to simultaneously multicast massive data streams to spatially distinct nodes with femtosecond‐scale latency and without active switching directly addresses the scalability and determinism requirements of distributed edge intelligence.

## 4 Conclusion

This study demonstrated an effective approach to multicast massive CVB signals within the volumetric Fresnel space using a 3D vector array as information carrier. Based on the principle of spin‐multiplexed phase modulation, a VDVG and a VDVZP were designed to generate multichannel, multimode CVBs across the 2D transverse plane and 1D axial depth. By integrating both elements into a spin‐multiplexed Dammann vortex metasurface, a 3D CVB array generator with spatially varying polarization topology distribution was obtained, experimentally demonstrating an array operational dimension of 3 × 3 × 5. To showcase its applicability in spatial multinode signal multicasting interconnection, we developed a CVB multicasting communication system and demonstrated the successful multicasting of 400 Gbit/s QPSK signals to eight or nine distinct receiver ports, achieving BERs on the order of 10^−6^. Our achievement highlights the ability of the proposed strategy to volumetrically tailor diverse CVBs within a compact minimalist metadevice, which may inspire further multidimensional optical field manipulation and create new opportunities for advanced communication networking and complex‐node interconnections.

## Author Contributions

**Zhiqiang Xie:** conceptualization, methodology, investigation, validation, writing – original draft, data curation, formal analysis. **Qingji Zeng:** conceptualization, methodology, data curation, investigation, formal analysis, visualization, writing – original draft, software. **Huihua Huang:** conceptualization, formal analysis, data curation, methodology, visualization, writing – original draft, software. **Zhibin Wu:** visualization, validation, data curation. **Kai Niu:** data curation, validation, visualization. **Junmin Liu:** funding acquisition, supervision, writing – review and editing, resources. **Shuqing Chen:** supervision, resources, writing – review and editing, funding acquisition. **Dianyuan Fan:** funding acquisition, writing – review and editing, supervision, resources. **Dingyuan Tang:** resources, supervision, writing – review and editing, funding acquisition.

## Funding

This work was supported by the National Natural Science Foundation of China (62271322, 62275162), Cui Can Program of Guangdong Province (202402BJ0602), Guangdong Basic and Applied Basic Research Foundation (2023A1515030152, 2025A1515011577), Shenzhen Science and Technology Program (JCYJ20240813143018024, JCYJ20241202124408012), and the National Science Foundation of Top Talent of SZTU (GDRC202302).

## Consent

Informed consent was obtained from all individuals included in this study.

## Conflicts of Interest

The authors declare no conflicts of interest.

## Supporting information


Supporting Information S1

## Data Availability

The data that support the findings of this study are available from the corresponding author upon reasonable request.

## References

[1] N. Chukhno , O. Chukhno , S. Pizzi , A. Molinaro , A. Iera , and G. Araniti , “Approaching 6G Use Case Requirements With Multicasting,” IEEE Communications Magazine 61, no. 5 (2023): 144–150, 10.1109/MCOM.001.2200659.

[2] R. Ma , J. Cao , Y. Zhang , C. Shang , L. Xiong , and H. Li , “A Group‐Based Multicast Service Authentication and Data Transmission Scheme for 5G‐V2X,” IEEE Transactions on Intelligent Transportation Systems 23, no. 12 (2022): 23976–23992, 10.1109/TITS.2022.3197767.

[3] N. Chukhno , O. Chukhno , D. Moltchanov , et al., “Models, Methods, and Solutions for Multicasting in 5G/6G mmWave and Sub‐THz Systems,” IEEE Communications Surveys & Tutorials 26, no. 1 (2024): 119–159, 10.1109/COMST.2023.3319354.

[4] J. Wang , J. Liu , S. Li , Y. Zhao , J. Du , and L. Zhu , “Orbital Angular Momentum and Beyond in Free‐Space Optical Communications,” Nanophotonics 11, no. 4 (2022): 645–680, 10.1515/nanoph-2021-0527. PMC11501694 39635376

[5] B. Chen , Y. Zhao , H. Tan , X. Guan , and S. Gao , “On‐Chip all‐Optical Multicasting of Mode‐Division Multiplexing QPSK Signals,” Electronics Letters 59, no. 15 (2023): e12903, 10.1049/ell2.12903.

[6] M. Tarhani , S. Sarkar , M. K. Eghbal , and M. Shadaram , “Efficient Multicasting Technique for Elastic Optical Network,” IET Networks 10, no. 1 (2021): 34–42, 10.1049/ntw2.12003.

[7] Z. Fan , Z. Yang , J. Dong , W. Dong , J. Xu , and X. Zhang , “Unlocking Multidimensional Optical Multicasting Based on Multi‐Mode PIN Silicon Waveguides,” Photonics Research 12, no. 12 (2024): 2938–2947, 10.1364/PRJ.536785.

[8] K. Keykhosravi , H. Rastegarfar , N. Peyghambarian , and E. Agrell , “Overcoming the Switching Bottlenecks in Wavelength‐Routing, Multicast‐Enabled Architectures,” Journal of Lightwave Technology 37, no. 16 (2019): 2569–2579, 10.1109/JLT.2019.2921679.

[9] Q. Zhan , “Cylindrical Vector Beams: From Mathematical Concepts to Applications,” Advances in Optics and Photonics 1, no. 1 (2009): 1–57, 10.1364/AOP.1.000001.

[10] Z. Shi , X. Wang , Z. Zhang , P. Wang , and Y. Liu , “Ultra‐Fast and Universal Vector Mode Analyzer Based on 4D Jones Vector and Intensity‐Only Detection,” Laser & Photonics Reviews 19, no. 14 (2025): 2500004, 10.1002/lpor.202500004.

[11] C. Zhou , W. Liang , Z. Xie , et al., “Optical Vectorial‐Mode Parity Hall Effect: A Case Study With Cylindrical Vector Beams,” Nature Communications 15, no. 1 (2024): 4022, 10.1038/s41467-024-48187-3. PMC11091081 38740787

[12] Y. Shen , H. Wang , and S. Fan , “Free‐Space Topological Optical Textures: Tutorial,” Advances in Optics and Photonics 17, no. 2 (2025): 295–374, 10.1364/AOP.547634.

[13] J. Morgan , P. R. Ribič , F. Capotondi , et al., “Poincaré Beams From a Free Electron Laser,” Nature Photonics 19, no. 9 (2025): 946–951, 10.1038/s41566-025-01737-7.

[14] Q. Zeng , S. Chen , Z. Wu , et al., “Advances in Mode (De)Multiplexing Technologies via Circularly Symmetric Structured Light Beams,” Advanced Photonics Research 6, no. 10 (2025): 2500088, 10.1002/adpr.202500088.

[15] K. Pan , X. Wu , P. Li , et al., “Cylindrical Vector Beam Holography Without Preservation of OAM Modes,” Nano Letters 24, no. 22 (2024): 6761–6766, 10.1021/acs.nanolett.4c01490. 38775803

[16] H.‐L. Tseng , C.‐Y. Han , K.‐H. Chen , C.‐H. Yeh , and J.‐H. Chen , “Recording of a Holographic Cylindrical Vector Beam Converter With a Truncated Cone Prism,” Optics Letters 47, no. 15 (2022): 3888–3891, 10.1364/OL.463323. 35913339

[17] Q. Zhao , S. Tu , Q. Lei , C. Guo , Q. Zhan , and Y. Cai , “Creation of Cylindrical Vector Beams Through Highly Anisotropic Scattering Media With a Single Scalar Transmission Matrix Calibration,” Photonics Research 10, no. 7 (2022): 1617–1623, 10.1364/PRJ.457928.

[18] Z. Yu , X. Gao , J. Yao , et al., “A Spatial‐Frequency Patching Metasurface Enabling Super‐Capacity Perfect Vector Vortex Beams,” eLight 4, no. 1 (2024): 21, 10.1186/s43593-024-00077-3.

[19] J. Liu , Z. Wu , Q. Zeng , et al., “Efficient Dynamic Free‐Space‐to‐Fiber Coupling of Cylindrical Vector Beams via Twisted Moiré Meta‐Devices,” Communications Physics 8, no. 1 (2025): 409, 10.1038/s42005-025-02323-7.

[20] J. Li , D.‐Y. Choi , J. T. Kim , and S. S. Lee , “Cylindrical Vector Beam Multiplexing Based on All‐Dielectric Fiber Meta‐Tips Enabling High‐Capacity Bidirectional Optical Communication,” Laser & Photonics Reviews 19, no. 24 (2025): e00738, 10.1002/lpor.202500738.

[21] Z. Xie , J. Liu , X. Wang , et al., “Cross‐Connection of Multiplexed Cylindrical Vector Beams Using Off‐Axis Spin‐Decoupled Metasurfaces,” Optics Letters 49, no. 10 (2024): 2533–2536, 10.1364/OL.520388. 38748098

[22] J. Zhang , Z. Ye , J. Yin , L. Liang , and S. Jiao , “Polarized Deep Diffractive Neural Network for Sorting, Generation, Multiplexing, and De‐Multiplexing of Orbital Angular Momentum Modes,” Optics Express 30, no. 15 (2022): 26728–26741, 10.1364/OE.463137. 36236859

[23] W. Meng , D. Pi , B. Li , H. Luan , M. Gu , and X. Fang , “Engineered 3D Vortex Array With Customized Energy and Topological Charges for Multi‐View Display,” Laser & Photonics Reviews 18, no. 10 (2024): 2301258, 10.1002/lpor.202301258.

[24] B. Zhao , R. Huang , and G. Lü , “Micro‐Projection Dynamic Backlight for Multi‐View 3D Display,” Chinese Optics Letters 19, no. 9 (2021): 092201, 10.3788/COL202119.092201.

[25] J. Chu , D. Chu , and Q. Smithwick , “Encoding and Multiplexing of 2D Images With Orbital Angular Momentum Beams and the Use for Multiview Color Displays,” Research: Ideas for Today’s Investors 2019 (2019): 9564593, 10.34133/2019/9564593. PMC6750116 31549097

[26] M. Liu , P. Lin , P. Huo , et al., “Monolithic Silicon Carbide Metasurfaces for Engineering Arbitrary 3D Perfect Vector Vortex Beams,” Nature Communications 16, no. 1 (2025): 3994, 10.1038/s41467-025-59234-y. PMC12041508 40301367

[27] P. Fu , P.‐N. Ni , Q.‐H. Wang , et al., “Multichannel Generations of Orbital Angular Momentum Modes With on‐Demand Characteristics on a Chip,” Advanced Optical Materials 9, no. 24 (2021): 2101308, 10.1002/adom.202101308.

[28] Q. Li , C. Wu , Z. Zhang , et al., “High‐Purity multi‐mode Vortex Beam Generation With Full Complex‐Amplitude‐Controllable Metasurface,” IEEE Transactions on Antennas and Propagation 71, no. 1 (2022): 774–782, 10.1109/TAP.2022.3217192.

[29] C. Zhou , Y. Zheng , G. He , et al., “Generating Arrays of Perfect Vector Vortex Beam With Six on‐Demand Degrees of Freedom in Metasurfaces,” Science China Physics, Mechanics & Astronomy 68, no. 5 (2025): 254211, 10.1007/s11433-024-2609-9.

[30] S. Fu , Y. Zhai , H. Zhou , et al., “Demonstration of Free‐Space One‐to‐Many Multicasting Link From Orbital Angular Momentum Encoding,” Optics Letters 44, no. 19 (2019): 4753–4756, 10.1364/OL.44.004753. 31568434

[31] P. Zhou , Q. Ji , X. Wen , et al., “Generation of Vectorial Optical Fields With Polarization Variation Along Propagation Paths via Dielectric Metasurfaces,” Nano Letters 25, no. 24 (2025): 1234–1241, 10.1021/acs.nanolett.5c02048. 40465374

[32] Z. Shang , S. Fu , L. Hai , Z. Zhang , L. Li , and C. Gao , “Multiplexed Vortex State Array Toward High‐Dimensional Data Multicasting,” Optics Express 30, no. 19 (2022): 34053–34063, 10.1364/OE.466353. 36242427

[33] J. Desai , K. K. Gangwar , Ruchi , K. Khare , and P. Senthilkumaran , “Generation of V‐Point Polarization Singularity Array by Dammann Gratings,” Applied Physics B 128, no. 6 (2022): 108, 10.1007/s00340-022-07830-x.

[34] J. Tao , Q. You , Z. Li , et al., “Mass‐Manufactured Beam‐Steering Metasurfaces for High‐Speed Full‐Duplex Optical Wireless‐Broadcasting Communications,” Advanced Materials 34, no. 6 (2022): 2106080, 10.1002/adma.202106080. 34825747

[35] X. Weng , L. Du , P. Shi , and X. Yuan , “Tunable Optical Cage Array Generated by Dammann Vector Beam,” Optics Express 25, no. 8 (2017): 9039–9048, 10.1364/OE.25.009039. 28437978

[36] Y. Tang , W. Perrie , J. Schille , et al., “High‐Quality Vector Vortex Arrays by Holographic and Geometric Phase Control,” Journal of Physics D: Applied Physics 53, no. 46 (2020): 465101, 10.1088/1361-6463/ab9d9b.

[37] Y.‐H. Zhang , P. Chen , S.‐J. Ge , et al., “Spin‐Controlled Massive Channels of Hybrid‐Order Poincaré Sphere Beams,” Applied Physics Letters 117, no. 8 (2020): 081101, 10.1063/5.0020398.

[38] W. Meng , B. Li , H. Luan , M. Gu , and X. Fang , “Orbital Angular Momentum Neural Communications for 1‐to‐40 Multicasting With 16‐ary Shift Keying,” ACS Photonics 10, no. 8 (2023): 2799–2807, 10.1021/acsphotonics.3c00500.

[39] S.‐J. Liu , P. Chen , S.‐J. Ge , L. Zhu , Y.‐H. Zhang , and Y.‐Q. Lu , “3D Engineering of Orbital Angular Momentum Beams via Liquid‐Crystal Geometric Phase,” Laser & Photonics Reviews 16, no. 6 (2022): 2200118, 10.1002/lpor.202200118.

[40] H. Yang , J. Xu , M. Peng , et al., “Vector Analog Computing via on‐Demand Metasurface Dispersive Polarization Transformation,” Science Advances 11, no. 42 (2025): eadz5123, 10.1126/sciadv.adz5123. PMC12525772 41091863

[41] N. Mahmood , Y. Park , N. Butt , et al., “Ultraviolet–Visible Spin‐Resolved Chip‐Scale Spectroscopy,” Advanced Functional Materials 35, no. 46 (2025): 2507112, 10.1002/adfm.202507112.

[42] Z. Xie , Z. Liang , H. Wu , et al., “Cylindrical Vector Beam Multiplexing Holography Employing Spin‐Decoupled Phase Modulation Metasurface,” Nanophotonics 13, no. 4 (2024): 529–538, 10.1515/nanoph-2023-0731. PMC11501858 39635652

[43] Q. Zeng , S. Liao , J. Chen , et al., “Helicity‐Decoupled Ge_2_Sb_2_Te_5_ Metasurfaces Enable Controllable Switching Between Single‐Layer and Cascaded Holograms,” Science China Technological Sciences 68, no. 7 (2025): 1720901, 10.1007/s11431-025-2944-x.

[44] X. Zhang , Y. Cui , Y. Chen , et al., “Full‐Parameter‐Modulated Three‐Dimensional Vectorial Generalized Vortex Array,” Light: Science & Applications 15, no. 1 (2026): 7, 10.1038/s41377-025-02065-9. PMC12756242 41476065

[45] Y. Hu , H. Chi , H. Duan , et al., “Metaoptics Merging Computational Optics and Optical Computing Toward Intelligent Visual Perception,” Science Advances 12, no. 1 (2026): eaea8941, 10.1126/sciadv.aea8941. PMC12758549 41481728

[46] L. Huang , X. Song , B. Reineke , et al., “Volumetric Generation of Optical Vortices With Metasurfaces,” ACS Photonics 4, no. 2 (2017): 338–346, 10.1021/acsphotonics.6b00808.

[47] Y. Zeng , H. Zhong , Z. Long , H. Cao , and X. Jin , “From Performance to Structure: A Comprehensive Survey of Advanced Metasurface Design for Next‐Generation Imaging,” npj Nanophotonics 2, no. 1 (2025): 39, 10.1038/s44310-025-00081-6.

[48] A. Parveen , A. Irshad , D. Tyagi , et al., “Design and Optimization of a Thermally Tunable Vanadium Dioxide‐Based Metasurface Optical Switch for Mid‐Infrared Applications,” Silicon 17, no. 14 (2025): 3391–3401, 10.1007/s12633-025-03425-1.

