# Supplementary material for: Three‐Dimensional Array Multicasting of Cylindrical Vector Beams Using Spin‐Multiplexed Dammann Vortex Metasurface
Source: Nanophotonics. 2026 Jul 15;15(14):e70215. doi: 10.1002/nap2.70215 (PMC13642607; doi:10.1002/nap2.70215)
Supplement: Supplementary file 1 [file NAP2-15-e70215-s001.docx]

Supplementary Information for

**Three-dimensional array multicasting of cylindrical vector beams using** **spin-multiplexed** **Dammann vortex metasurface**

Zhiqiang Xie^1,#^, Qingji Zeng^2,#^, Huihua Huang^2,#^, Zhibin Wu^1^, Kai Niu^1^, Junmin Liu^1,*^, Shuqing Chen^2^, Dianyuan Fan^2^ and Dingyuan Tang^1,*^

^1^Future Technology School, College of New Materials and New Energies, Shenzhen Technology University, Shenzhen 518118, China

^2^Institute of Microscale Optoelectronics, Shenzhen University, Shenzhen 518060, China

^#^These authors contributed equally to this work: Zhiqiang Xie, Qingji Zeng, Huihua Huang

^*^Corresponding authors: Junmin Liu ([liujunmin@sztu.edu.cn](mailto:liujunmin@sztu.edu.cn)) and Dingyuan Tang ([tangdingyuan@sztu.edu.cn](mailto:tangdingyuan@sztu.edu.cn))

__________________________________________________________________________

**Supplementary Note S1: Spin-multiplexed array synthesis and parameter design**

We present the engineering implementation of 3D CVB array through spin-multiplexed scalar vortex modulation. As theoretically established in Equation (1) of the main text, two binary scalar DVG phase profiles are derived to independently manipulate the orthogonal LHCP and RHCP components, resulting in 2D vortex array outputs with opposite spin-conjugated modes in the *x*-*y* transverse plane, as shown in Figure S1a. The phase-modulation matrix comprises a 1000×1000-pixel array with 800 μm pixel pitch. The phase periods along both orthogonal directions, namely Λ*_x_* and Λ*_y_*, are set to 10 μm for the DVG structure. To prove the generated orbital angular momentum states, the vortices at individual diffraction orders are physically characterized through interference with coherent spherical reference waves. The resulting spiral interferograms provide a direct readout of both the magnitude and sign of the topological charge: the number of spiral arms corresponds to the absolute topological charge, while the spiral handedness distinguishes its sign. Under our measurement configuration, counter-clockwise and clockwise spiral fringes correspond to negative and positive topological charges, respectively. As seen, the calculated interference patterns are in excellent agreement with the designed vortex-array distributions. Based on Equation (3) of the main text, a pair of spin-dependent binary scalar DVZP phases, combined with a shared lens phase, are solved to generate two independent 1D longitudinal vortex arrays along the axial propagation direction. The analytically derived phase distributions and the corresponding output intensity profiles in the *y*-*z* transverse plane are presented in Figure S1b. In the design of this element, the period Λ*_z_* of the DVZP is set to 0.325 nm, the parameter *α* to 0.2447, and the focal length of the lens phase is defined as 1.2 mm. As observed, the transverse light field profiles and spherical-wave interference patterns shown in the figure collectively verify that the generated vortex beams carry the expected topological charges, consistent with the designed vortex-array outputs. Synthesis of the two phase components upon individual spin states yields a composite profile that enables volumetric multi-channel multi-mode operations under Gaussian illumination, producing 3D diffractive vortex arrays in the Fresnel region, as reflected in Figure S1c. Apparently, diverse 2D array patterns are projected at five distinct spatial depths (*z*_1_, *z*_2_, *z*_3_, *z*_4_, *z*_5_), with their depth-dependent evolution spawning progressive 3D wavefront engineering. A spin-multiplexed strategy ultimately leads to the generation of a 3D cylindrical vector array, with findings described in Supplementary Note S3.

**FIGURE S1 |** Phase profiles for LHCP and RHCP components and the resulting evolution of array optical field. (a) Generation of 2D planar scalar vortex arrays using two spin-dependent DVG phases. (b) Formation of 1D axial scalar vortex arrays based on two spin-dependent DVZP phases. (c) Synthesized 3D scalar vortex arrays under axial diffraction propagation. Embedded white numerals in the output fields serve to characterize the topological charges inherent to these vortex beams.

**Supplementary Note S2: Fabrication of metasurface sample**

In this work, we used the standard electron-beam lithography (EBL) system to fabricate the proposed all-dielectric Si-SiO_2_ metasurface, and the corresponding fabrication flow is outlined in Figure S2. Frist of all, an 800-nm-thick Si film was deposited on a double-side-polished SiO_2_ substrate by the magnetron sputtering technology. Subsequently, a 150-nm-thick polymethyl methacrylate (PMMA, 950K) resist layer was spin-coated onto the Si-SiO_2_ substrate at 4000 rpm for 60 s, followed by baking at 180 °C for 90 s to solidify the resist. The desired Dammann vortex meta-patterns were then defined by EBL at an acceleration voltage of 20 keV. After exposure, the sample was developed in a 1:3 MIBK:IPA solution for 60 s and rinsed in IPA for 30 s to stop the development. Next, a 30-nm-thick chromium (Cr) layer was deposited using an electron beam evaporator, and lift-off was carried out in 30% acetone solution for 6 h to form the Cr hard mask. The patterns were transferred from the Cr mask into the Si layer using inductively coupled plasma reactive ion etching (ICP-RIE) with a mixed SF_6_/CHF_3_ gas chemistry (SF_6_:CHF_3_ = 1:7). Finally, the Cr mask on top of the Si meta-atom was removed by wet etching in a 1:2 solution of Ce(NH_4_)_2_(NO_3_)_6_:HNO_3_, and we can get the Si-SiO_2_ metasurface.

**FIGURE S2 |** Manufacturing procedures for the proposed all-dielectric transmissive Si-SiO_2_ Dammann vortex metasurface.

**Supplementary Note S3: Simulation demonstration of 3D CVB array**

Figure S3 demonstrates the simulated optical field of our designed 3D CVB array, including the corresponding total intensity and the *x*- and *y*-polarized detection results. The figure displays cross-sectional views of five transverse 3 × 3 arrays at distinct diffraction depths (*z*_1_ to *z*_5_) within the Fresnel range, thereby forming a 3 × 3 × 5 operational architecture for 3D CVB engineering. In the numerical simulations, the positions of the five transverse observation planes are set at diffraction distances of *z*_1_ = 710 μm, *z*_2_ = 889 μm, *z*_3_ = 1187 μm, *z*_4_ = 1775 μm, and *z*_5_ = 3530 μm.

**FIGURE S3 |** Simulation results of 3D CVB array comprising five transverse 3 × 3 sub-arrays across different *z*-axis propagation depths, presenting the corresponding (a) total intensity, (b) *x*-polarized, and (c) *y*-polarized detection outcomes. The white double arrow indicates the direction of polarization.

**Supplementary Note S4: Bandwidth evaluation of the 3D CVB array system**

In this section, we elucidate and quantitatively analyze the intrinsic bandwidth limitations of the proposed 3D CVB array system. This spectral constraint primarily stems from the wavelength-dependent nature of the phase modulation function (Equation (4) in the main text) tailored for 3D CVB engineering, particularly impacting the axial diffractive profiling. To benchmark this effect, we evaluated the transverse 2D array distributions at a fixed depth plane (*z*_1_ = 710 μm) under illumination by monochromatic Gaussian beams of varying wavelengths. The operational bandwidth was determined by quantifying the diffractive efficiency ratios (*Δ*) and assessing the focal spot profiles.

**FIGURE S4 |** Simulation results of the transverse 2D array distributions at a fixed depth plane of *z*_1_ = 710 μm. The blue box shows a magnified view of the focal spot profiles.

Figure S4 presents the simulated array distributions across 15 discrete wavelengths, maintaining a system configuration identical to that in Supplementary Note S3 except for the illumination wavelength. Deviations from the designated design wavelength of 1550 nm visibly induce light-field degradation. For shorter wavelengths (*λ* < 1550 nm), severe speckle noise emerges within the array fields, indicating a decline in diffractive modulation efficiency. Quantitatively, at *λ* = 1516 nm, the relative diffractive efficiency drops by 30.62% compared to the baseline at 1550 nm, with the efficiency rolling off further as the wavelength detuning increases. For longer wavelengths (*λ* > 1550 nm), the power uniformity among the multiple diffractive orders becomes severely imbalanced, accompanied by a progressive spatial distortion of the focal spots. Ultimately, under 1605 nm illumination, the diffractive orders fail to sustain their original Gaussian profiles, rendering them unsuitable for high-fidelity 3D CVB array generation. Consequently, by establishing a tolerance threshold of less than a *Δ* = 30% efficiency degradation alongside high-fidelity spot profile preservation, the effective operating bandwidth of the system is identified as 1517−1600 nm (a total span of 83 nm). While this bandwidth restriction is an intrinsic physical boundary of the diffractive architecture, this 83 nm spectral window remains sufficiently broad to accommodate numerous dense wavelength-division multiplexing (DWDM) channels. This robust spectral tolerance underscores the system’s capacity to seamlessly integrate into high-capacity, multi-channel WDM communication networks with substantial broadband response.

**Supplementary Note S5: System of 3D CVB array multicasting communication**

A dedicated prototype optical communication system was developed to validate the performance of the fabricated meta-device in 3D multicasting interconnection, comprising three main components: the transmitter, free-space CVB array multicasting link, and receiver, as schematically illustrated in Figure S5. At the transmitter terminal, four incoherent Gaussian laser lines at discrete wavelengths of 1548.52 nm, 1550.12 nm, 1551.72 nm, and 1553.33 nm are coupled into a single beam via a wavelength division multiplexer (WDM). Each wavelength channel is encoded with a 50 Gbit/s QPSK signal generated by a programmable pulse generator (PPG) and subsequently modulated using an in-phase/quadrature (IQ) modulator. A polarization division multiplexer (PDM) then doubles the channel capacity. After amplification by an erbium-doped fiber amplifier (EDFA) to boost the signal power, the total data rate entering the multicasting link reaches 400 Gbit/s (50 Gbit/s × 4 × 2).

At the free-space CVB array multicasting link, the fundamental Gaussian beam is collimated from the fiber into free space using a fiber collimator. After its initial polarization orientation is adjusted by a HWP and its size resized by a lens, the beam impinged on our metasurface-based multicasting device to achieve spatial volumetric replication and distribution of the signal streams. A Q-plate (indicated by the dashed box in the figure) is optionally employed to convert the input beam into the orthogonally polarized CVB modes, whose polarization order are determined by the selected *q*-value of the Q-plate, thereby serving as the input channel source. Within the diffracted 3D CVB lattice, only the diffraction orders matching the conjugate of the input CVB mode can be retrieved back to Gaussian beams, serving as the multicasting signal access port for the corresponding node. Therefore, the optical identification and detection process is passive and instantaneous: the presence of a Gaussian beam at a specific spatial node directly indicates that the incoming CVB had the conjugate polarization order. No active measurement or computational reconstruction of the polarization order is needed; the detection is simply based on the intensity of the recovered Gaussian spot. These Gaussian spots are subsequently magnified by an OL and selectively filtered by an iris to isolate the desired multicasting output nodes, after which the signal flow is directly coupled into standard single-mode fiber via a fiber collimator without requiring any ancillary or active mode-conversion hardware.

At the receiver terminal, the received signals are wavelength-demultiplexed by a wavelength division demultiplexer (De-WDM), polarization-selected via a polarization beam splitter (PBS), and power-adjusted using an EDFA in turn. After coherent demodulation in an integrated coherent receiver (ICR), the signals are sampled with an oscilloscope for offline digital signal processing (DSP), and the resulting data are used to evaluate the communication performance. Notably, the spin‑multiplexed Dammann vortex metasurface is a passive optical element operating at the speed of light. The “encoding” of the CVB array and the “decoding” back to Gaussian beams occur via diffraction, which is an instantaneous process governed by the propagation of electromagnetic waves. There is no electronic or mechanical switching involved; the transformation from input CVB to output 3D array and then to Gaussian spots at specific nodes happens within the transit time of light through the metasurface (on the order of femtoseconds). Therefore, the metasurface itself does not impose any measurable latency or processing time. The 400 Gbit/s data rate refers to the modulation rate of the QPSK signals carried by the optical carrier, not to the switching speed of the metasurface. In our system, the metasurface simultaneously multicasts the entire high‑speed data stream to all output nodes without any time‑division multiplexing or serial processing, ensuring that the full 400 Gbit/s throughput is available at each access port.

**FIGURE S5 |** Schematic of experimental prototype system for 3D array multicasting communication. IQ Mod., in-phase/quadrature modulator; Col., collimator; MS, metasurface; VOA, variable optical attenuator; LO, local oscillator.

**Supplementary Note S6: Measured waveforms in signal multicasting**

To validate the one-to-many data replication capability of our multicasting scheme, we demonstrate the case of 9-channel data allocation under 0-polarization-order CVB incidence. Figure S6 presents the captured temporal waveforms of the input source and the corresponding post-multicast receiver channels. The preserved temporal waveforms across all channels are accordingly maintained, underscoring the successful signal dissemination enabled by CVB array multicasting.

**FIGURE S6 |** Observed demodulated waveforms of in-phase components of input source and the post-multicasting receiver channels.

**Supplementary Note S7: Extended multicasting communication findings**

To validate the robustness and versatility of the proposed 3D CVB array multicasting system, we further investigated the communication performance under the excitation of higher-order CVBs with polarization orders of ±4. The experimentally retrieved bit error rate (BER) curves and corresponding constellation diagrams are illustrated in Figure S7. As depicted in the BER evolution profiles, the back-converted Gaussian access ports exhibit a monotonic decay in BER orders as the received optical power scales up. Notably, the observed channel-to-channel BER variations primarily arise from the non-uniform energy distribution among diffraction orders, residual mode impurities from imperfect polarization conversion, wavelength-dependent power imbalances across the wavelength-division multiplexing channels and EDFA gain profiles, as well as minor experimental misalignments and inter-order scattering interference. Despite these subtle discrepancies, the sampled constellation diagrams of selected channels exhibit highly consistent and convergent profiles; at a received optical power of −18 dBm, all corresponding error vector magnitudes (EVMs) remain strictly below 0.229. Synthesizing these findings with the baseline data in Figure 5 of the main text reveals that injecting CVBs with different polarization orders dictates that different sets of diffraction orders are retrieved as Gaussian access ports due to the inherent conjugate-matching condition, thereby resulting in distinct spatial configurations for the multicasting receiver ports. Crucially, despite this topology-dependent port allocation, the overall BER performance remains highly comparable across all tested orders, with all operational channels comfortably residing well below the hard-decision forward error correction (FEC) threshold (3.8 × 10^−3^). These results unambiguously demonstrate that the system preserves a robust, order-invariant transmission fidelity, ensuring consistent broadband multicasting capacity regardless of the input modal variations.

**FIGURE S7 |** Communication results of data dissemination using 3D CVB array multicasting. Measured BER curves and constellation diagrams for signal multicasting with (a) +4-polarization-order and (b) −4-polarization-order CVB input sources.

**Supplementary Note S8: Scalability demonstration and characterization of the 5 × 5 × 5 CVB array**

To underscore the scalability and architectural potential of our 3D CVB array generation methodology, the array dimensionality was further expanded to a volumetric 5 × 5 × 5 configuration. As illustrated in Figure S8, the array is scaled by expanding the 2D planar transverse dimensions while maintaining the identical 1D longitudinal axial configuration as the baseline 3 × 3 × 5 format. Because the formulated 3D CVB lattice originates from the mathematically orthogonal and multiplicative synthesis of a 2D planar array and a 1D axial array, optimizing the phase transfer function for the 2D in-plane array (i.e., Equation (2) in the main text) leaves the longitudinal diffraction distribution unperturbed. Consequently, the spatial positions of the five axial depth planes remain invariant at *z*_1_ = 710 μm, *z*_2_ = 889 μm, *z*_3_ = 1187 μm, *z*_4_ = 1775 μm, and *z*_5_ = 3530 μm. This transverse scaling is flexibly realized by tuning the design parameters to *M* = *N* = 4 within the transfer function, thereby yielding a deterministic (*M* + 1) × (*N* + 1) = 5 × 5 transverse CVB array. Crucially, the embedded Dammann grating optimization inherently guarantees a strict centrosymmetric conjugate symmetry for the polarization orders, effectively eliminating any structural and polarimetric asymmetries along the horizontal and vertical axes. This polarization invariance is rigorously verified by the modal characterizations of the multiple diffracted CVB sub-beams in Figure S8, a property that ensures deterministic, polarization-distortion-free channel allocation across the massive network.

Although distributing the total incident power among a significantly larger number of nodes inevitably leads to a lower per-channel received power, the Dammann architecture preserves a remarkably balanced energy distribution among all active channels. This energy equalization may guarantee that the multicast signals maintain uniform signal-to-noise ratios (SNRs) across all receiving ports, projecting a tightly clustered and homogeneous BER distribution under identical received power levels. Predictably, while splitting the incident optical power among such large-scale multicasting nodes reduces the signal strength allocated to individual receiver channels—which could theoretically introduce vulnerability to free-space atmospheric turbulence and impact single-mode fiber coupling efficiency—the overall transmission fidelity remains highly robust. Owing to the sharp spatial isolation and negligible inter-channel cross-talk between the adjacent diffracted spots, any power-induced SNR degradation can be easily mitigated by scaling the initial input pump power, which is poised to validate the system’s viability for massive, high-capacity 3D parallel optical interconnections.

**FIGURE S8 |** Simulated diffraction patterns of a 5 × 5 × 5 operation-dimensional 3D CVB array. Total intensity, *x*-polarized, and *y*-polarized components are presented. The white double arrow denotes the polarization orientation.
